# RE: Evaluating the impact of virtual oncology financial and legal navigation on cancer-related financial toxicity

**DOI:** 10.1093/jncics/pkag093

**Published:** 2026-09-12

**Authors:** Lucio Buffoni

**Affiliations:** S.C. Oncologia ed Ematologia Oncologica, Ospedale Regionale Umberto Parini, Azienda USL Valle d‘Aosta, Aosta, Italy

Edward et al. provide timely evidence that virtual oncology financial and legal navigation may mitigate cancer-related financial toxicity, particularly among caregivers and patients facing geographic barriers to specialized cancer care. Their findings also raise a broader question: how transferable are financial navigation interventions across different healthcare systems?^1^

Financial toxicity is not a uniform construct. Recent European evidence highlights substantial socioeconomic consequences of cancer even where universal or predominantly publicly funded healthcare limits direct treatment costs.^2^ A systematic review specifically examining publicly funded healthcare systems similarly showed persistent financial burden related to lost income, travel and accommodation, out-of-pocket expenditure, and caregiver costs.^3^ Thus, universal coverage does not eliminate financial toxicity; it changes its composition.

This distinction is increasingly relevant. A recent global meta-analysis found that 46% of the heterogeneity in Comprehensive Score for Financial Toxicity estimates was attributable to between-country differences, with macroeconomic and structural factors significantly associated with financial burden.^4^ Financial toxicity should therefore be considered partly a property of the healthcare and socioeconomic environment in which cancer occurs, rather than solely a patient-level outcome.

This has implications for digital navigation. In universal healthcare systems such as the Italian National Health Service, effective virtual navigation might focus less on insurance-related assistance and more on access coordination, social benefits, employment protection, transportation and accommodation, informal caregiving, and reduction of avoidable hospital visits. This is particularly relevant for patients living in rural, mountainous, or otherwise underserved areas, where indirect costs may become substantial despite publicly funded cancer treatment.

Accordingly, the value of virtual navigation should not be assessed solely by whether a global financial toxicity score improves. Implementation studies should determine which components of financial toxicity are modified, for whom, and through which mechanisms. This is particularly important because financial toxicity remains heterogeneous across healthcare systems,^5^ although screening approaches themselves have not yet been standardized for routine clinical practice.^6^

The digital component also deserves scrutiny. Virtual navigation may reduce geographic barriers, but differences in digital access and literacy could generate new inequalities. Future studies should therefore complement financial toxicity measures with implementation outcomes including reach, uptake, equity, resource utilization, scalability, and integration with existing oncology pathways.

The study by Edward et al. provides an important proof of concept. The next step may be to move from asking whether virtual financial navigation works to understanding how navigation should be redesigned according to the healthcare system in which cancer care is delivered. Financial toxicity is system-dependent; interventions intended to mitigate it should be equally sensitive to context.

## Data Availability

No new data were generated or analyzed for this correspondence.
